# The management of ADHD in children and adolescents: bringing evidence to the clinic: perspective from the European ADHD Guidelines Group (EAGG)

**DOI:** 10.1007/s00787-021-01871-x

**Published:** 2021-10-22

**Authors:** David Coghill, Tobias Banaschewski, Samuele Cortese, Philip Asherson, Daniel Brandeis, Jan Buitelaar, David Daley, Marina Danckaerts, Ralf W. Dittmann, Manfred Doepfner, Maite Ferrin, Chris Hollis, Martin Holtmann, Santosh Paramala, Edmund Sonuga-Barke, César Soutullo, Hans-Christoph Steinhausen, Saskia Van der Oord, Ian C K Wong, Alessandro Zuddas, Emily Simonoff

**Affiliations:** 1 Faculty of Medicine, Dentistry and Health Sciences, University of Melbourne, Melbourne, Australia; 2 Murdoch Children’s Research Institute, Melbourne, Australia; 3 Royal Children’s Hospital, Melbourne, Australia; 4 Child and Adolescent Psychiatry and Psychotherapy, Medical Faculty Mannheim, Central Institute of Mental Health, University of Heidelberg, Mannheim, Germany; 5 Centre for Innovation in Mental Health, School of Psychology, Faculty of Environmental and Life Sciences, University of Southampton, Southampton, UK; 6 Clinical and Experimental Sciences (CNS and Psychiatry), Faculty of Medicine, University of Southampton, Southampton, UK; 7 Clinical and Experimental Sciences (CNS and Psychiatry), Faculty of Medicine, University of Southampton, Southampton, UK; 8 Solent NHS Trust, Southampton, UK; 9 Hassenfeld Children’s Hospital at NYU Langone, New York University Child Study Center, New York City, New York USA; 10 Division of Psychiatry and Applied Psychology, School of Medicine, University of Nottingham, Nottingham, UK; 11 NIHR MindTech Mental Health MedTech Cooperative & Centre for ADHD and Neurodevelopmental Disorders Across the Lifespan CANDAL, Institute of Mental Health, University of Nottingham, Nottingham, UK; 12 Social, Genetic and Developmental Psychiatry Centre, Institute of Psychiatry, Psychology and Neuroscience, King’s College London, London, UK; 13 Department of Child and Adolescent Psychiatry and Psychotherapy, Psychiatric Hospital, University of Zurich, Zurich, Switzerland; 14 Neuroscience Center Zurich, University of Zurich and ETH Zurich, Zurich, Switzerland; 15 Center for Integrative Human Physiology, University of Zurich, Zurich, Switzerland; 16 Department of Cognitive Neuroscience, Donders Institute for Brain, Cognition and Behaviour, Radboud University Medical Center, Nijmegen, The Netherlands; 17 Research Group of Developmental Psychiatry, Center for Developmental Psychiatry, KU Leuven, Kapucijnenvoer 7, Blok H, 3000 Leuven, Belgium; 18 Department of Child and Adolescent Psychiatry, UPC KU Leuven, Leuven, Belgium; 19 Paediatric Psychopharmacology, Department of Child and Adolescent Psychiatry and Psychotherapy, Central Institute of Mental Health, Medical Faculty Mannheim, University of Heidelberg, Heidelberg, Germany; 20 Department of Child and Adolescent Psychiatry, Psychosomatics and Psychotherapy, Faculty of Medicine, University Hospital Cologne, University of Cologne, Cologne, Germany; 21 Haringey CAMHS, NHS, and ReCognition Health, London, UK; 22 LWL-University Hospital for Child and Adolescent Psychiatry, Ruhr-University Bochum, Hamm, Germany; 23 Department of Child and Adolescent Psychiatry, Institute of Psychiatry, Psychology and Neuroscience, King’s College London, London, UK; 24 Maudsley Biomedical Research Centre, South London and Maudsley NHS Foundation Trust, London, UK; 25 Department of Child and Adolescent Psychiatry, Aarhus University, Aarhus, Denmark; 26 Louis A Fallace Department of Psychiatry and Behavioral Science, University of Texas, Houston, TX USA; 27 Department of Child and Adolescent Psychiatry and Psychotherapy, University Hospital of Psychiatry, Zurich, Switzerland; 28 Clinical Psychology and Epidemiology, Institute of Psychology, University of Basel, Basel, Switzerland; 29 Department of Child and Adolescent Mental Health, University of Southern Denmark, Odense, Denmark; 30 Child and Adolescent Mental Health Centre, Capital Region Psychiatry, Copenhagen, Denmark; 31 Clinical Psychology, KU Leuven, Leuven, Belgium; 32 Developmental Psychology, University of Amsterdam, Amsterdam, The Netherlands; 33 School of Pharmacy, University College London, London, UK; 34 Child and Adolescent Neuropsychiatry Unit, Department of Biomedical Sciences, University of Cagliari and Antonio Cao Paediatric Hospital, G. Brotzu Hospital Trust, Cagliari, Italy

**Keywords:** Attention deficit hyperactivity disorder, Intervention, Evidence-based medicine, Guideline

## Abstract

ADHD is the most common neurodevelopmental disorder presenting to child and adolescent mental health, paediatric, and primary care services. Timely and effective interventions to address core ADHD symptoms and co-occurring problems are a high priority for healthcare and society more widely. While much research has reported on the benefits and adverse effects of different interventions for ADHD, these individual research reports and the reviews, meta-analyses and guidelines summarizing their findings are sometimes inconsistent and difficult to interpret. We have summarized the current evidence and identified several methodological issues and gaps in the current evidence that we believe are important for clinicians to consider when evaluating the evidence and making treatment decisions. These include understanding potential impact of bias such as inadequate blinding and selection bias on study outcomes; the relative lack of high-quality data comparing different treatments and assessing long-term effectiveness, adverse effects and safety for both pharmacological and non-pharmacological treatments; and the problems associated with observational studies, including those based on large national registries and comparing treatments with each other. We highlight key similarities across current international clinical guidelines and discuss the reasons for divergence where these occur. We discuss the integration of these different perspective into a framework for person/family-centered evidence-based practice approach to care that aims to achieve optimal outcomes that prioritize individual strengths and impairments, as well as the personal treatment targets of children and their families. Finally, we consider how access to care for this common and impairing disorder can be improved in different healthcare systems.

## Introduction

Attention deficit/hyperactivity disorder (ADHD) is a neurodevelopmental disorder defined by persistent, impairing and developmentally inappropriate inattentive/disorganized and/or hyperactive/impulsive behaviors that lie at the far end of a normally distributed continuum. [1] ADHD is common, with a worldwide pooled prevalence of around 5.3% in children and adolescents [2] and 2.8% in adults [3]. Although epidemiological data suggest that, when the same diagnostic criteria and procedures are applied, epidemiological prevalence rates of ADHD are similar throughout the world, the administrative prevalence for ADHD (rate of clinical diagnosis) varies considerably both between and within countries [4].

ADHD results in considerable burden at an individual, family, and societal level and has an important impact on quality of life and daily functioning [5, 6]. Patients with ADHD are at increased risk for serious negative outcomes including poor educational outcomes [7], injuries and accidents [8, 9], teenage pregnancies [10], family conflict [11], and criminal behavior and incarceration [12]. ADHD is also commonly associated with other psychiatric and neurodevelopmental disorders [13–20] and a number of physical health conditions [21–23]. A recent Australian study (population 24.6 million) [24] estimated the total costs in 2019 as $20.57 billion AUD (£11.3 billion UKP, €12.9M,), which translates to $836 AUD (£459 UKP, €524) per capita. Of this, total 63% were attributable to financial costs and 37% to well-being costs (those costs associated with reduced quality of life and impaired functioning, and premature death).

While there is now considerable evidence to support the efficacy and safety, at least in the short term, of pharmacological [25] and some non-pharmacological treatments [26] for ADHD, there are also indications that the positive effects seen in clinical trials are not always realized in day-to-day clinical practice [27, 28]. The purpose of this paper is to critically discuss the most up-to-date clinical evidence on the potential benefits and harms of the various approaches to the treatment and management of ADHD, and to identify the limitations of the current evidence base and the impact of these limitations on interpretation and translation into clinical practice.

This work did not involve primary research, and therefore, no ethical approval was required. The focus is on children and adolescents, because a range of both pharmacological and non-pharmacological interventions has been evaluated in this age range, while the evidence in adults is limited largely to pharmacological interventions. While most clinical trial data have focused on core ADHD symptoms as the primary outcome of interest, an increasing number of studies have recognized the importance of a broader range of outcomes. These include common co-occurring symptoms such as mood lability and those related to coexisting disorders (i.e., ODD, anxiety and depression)[29], functional impairments, quality of life as and more distal outcomes such as criminality [30], traffic accidents [31], and mortality rates [32]. We follow this lead on outcome beyond core symptoms in the current paper. As ADHD is usually a chronic and long-term condition [33], we will wherever possible consider evidence that focuses on long-term as well as short-term outcomes.

We will first consider some of the methodological challenges that we encountered when conducting systematic reviews and meta-analyses of ADHD interventions and highlight how these may have affected interpretation of the evidence and discuss some of the approaches that may mitigate these methodological challenges. We then compare different sets of guidelines in relation to ADHD management, with a particular focus on the common features and highlighting important differences, including possible reasons for these discrepancies. We then discuss how the evidence and guidelines can be translated into high-quality care, and how the clinician can take account of individual symptom profiles, treatment targets, and personal circumstances to provide person- and family-centered approaches to intervention within the current evidence base. Finally, we consider the implications for systems of care and how adherence to evidence-based practice can be implemented across a range of care settings.

## The evidence base for interventions

### Methodological issues

In this section, summarized in Table 1, we highlight several key methodological issues, consider their potential impact on evidence-based decision-making, and give some guidance on what aspects to look for when assessing the evidence.

**Table 1:** Methodological issues relating to current evidence base for assessing ADHD treatments (pharmacological and non-pharmacological)

Methodological problem	Impact of this on research	Impact on clinical decision-making	Potential solutions
Adequacy of blinding			
Potential for unblinding in pharmacological studies due to adverse effects or dramatic positive effects in active treatment arm	May inflate the apparent effects of active treatments	Reduces confidence in evidence-based recommendations	Potential for two independent raters, one for efficacy and one for side effect?
Inadequate blinding in psychosocial interventions particularly where patients and/or their families directly involved in the treatment provide the information about outcomes	Inflates effects of treatment	If not considered can lead to overconfident treatment recommendations	Researchers should make greater efforts to include blinded ratings in clinical trials of psychosocial interventions with the optimal approach using a combination of blinding by design and blinding by reporter. When reviewing evidence from studies in which a range of outcomes have been used, reviewers should consider adequacy of blinding. Consider adopting the innovative approach developed by the EAGG which compares the most proximal outcome (MPROX—i.e., the outcome provided by the rater closest to treatment delivery and therefore the most vulnerable to this effect of lack of blinding) with the measure judged to be most blinded (PBLIND)
Trial design			
The use of pre-specified outcomes in statistical analysis plan	A failure to include, and stick to, pre-specified outcomes when analyzing the results of a trial can lead to biases in reporting. These biases will usually result in over estimation of treatment effects through ‘cherry picking’ of the most favorable outcomes	Overestimation of treatment effects and overconfidence in clinical decision-making	Ensure that all primary and secondary outcomes are pre-specified in a comprehensive statistical analysis plan at trial registration and that this is published/made publicly available well before recruitment is completed. Ensure that the statistical analysis plan is adhered to and that when reporting results any deviations from the plan or additional are clearly and accurately reported as such in trial reports and publications
Trial selection criteria			
The use of enriched sample designs, e.g., by excluding participants who have previously not responded to or had adverse effects from one of the study drugs	Although these requirements were introduced for ethical reasons, they are likely to produce biased results, usually in favor of the drug compared to either placebo or an active comparator	Reduces confidence in evidence-based recommendations	Ensure adequate reporting of trial design in reports and inclusion of potential biases in limitation sections. Consider excluding studies with enriched designs from meta-analyses and network meta-analyses
Lack of long-term efficacy, effectiveness, and safety data for pharmacological treatments			
High-quality data on long-term outcomes is still lacking and the long-term effectiveness of ADHD medications continues to be debated	Limits the strength of recommendations about efficacy and effectiveness of long-term medication use for ADHD	A lack of evidence for long- term efficacy is not the same as evidence for ineffectiveness. Evidence suggests that the use of measurement-based care can lead to long-term effectiveness	The use of innovative trial designs including randomised discontinuation trials (REFS) that can evaluate effectiveness over longer time periods The use of linked registry data and data from electronic medical records to assess longer term outcomes in real-world situations
Lack of accurate data on the potential adverse effects of long-term treatment with ADHD medications	Adverse events are traditionally measured through spontaneous self-report and over relatively short time frames Recent post-marketing studies for new ADHD medications have extended the time-frame up to 2 years and included structured assessments but have not yet included a comparison group	While the long-term (up to 16 years) data from the MTA study do highlight probably effects of stimulant medications on growth (REF), this, and other recent longer term studies have not identified other serious group level growth and puberty, cardiovascular, psychiatric or neurological adverse effects	The use of innovative study designs such as… using large cohorts, linked registry data and data from electronic medical records that include well matched comparison groups of unmedicated ADHD participants as well as health individuals
Lack of data on potential adverse effects associated with non-pharmacological ADHD treatments			
Very few trials of non-pharmacological treatments for ADHD have included measures of adverse effects	The assumption that such interventions are unlikely to have any negative effects is perhaps naive and certainly untested	Clinicians should consider the potential adverse effects that may be associated with non-pharmacological treatments (e.g., potential for parental conflict during parent training; adverse effects from dietary manipulation)	Clinical trials of non-pharmacological treatments should routinely include measures of adverse effects
Network meta-analyses			
Transitivity assumption	If not respected, results of NMA are not valid	NMAs may support an inaccurate ranking of treatments	Include in the NMA only studies that are similar in terms of effect modifiers (demographic/clinical/methodological characteristics)
Including pharmacological and non-pharmacological interventions in the same network	Results may be misleading	One type of treatment (e.g., pharmacological or non-pharmacological) may erroneously be favored over the other	Test if control conditions have similar effects before lumping them in the same node; check differences between pharmacological and non-pharmacological studies in patient characteristics; assess the association of high risk of bias for lack of masking with the treatment effects; use Component NMAs and/or individual Patient Data (IPD)-NMAs

### Evaluating the quality of evidence: risk of bias

Randomized-controlled trials (RCTs) remain the gold standard to assess the efficacy, effectiveness, and safety of interventions. In the last couple of decades, there has been a greater appreciation of the importance of rigorous trial design and analyses to ensure that treatment evaluations are not biased. Standardized instruments, such as the Cochrane Risk of Bias tool [34] and GRADE [35] criteria, have been applied to assess potential bias of individual trials and the overall quality of the evidence, respectively. Different review groups have applied these tools differently and as a consequence come to different conclusions about the overall quality of evidence and how it should be interpreted (e.g., [25, 36]). These differences often reflect the way thresholds are set (e.g., number of ‘uncertain’ items required for a study to be rated as ‘high risk’) or different approaches to evaluate the impact of potential conflicts of interest. Unlike considerations for pharmacological interventions, there has, until recently, been little attempt to take account of researchers’ involvement in both the development and evaluation of non-pharmacological interventions as an area of potential risk of bias (but see [37] for a recent advance in the field). In the UK, the National Institute for Healthcare and Excellence (NICE) recently rated the overall quality of evidence as *low* for non-pharmacological approaches and *low-to-moderate* for pharmacological interventions [38]. Of note, the evidence level for pharmacological interventions is comparable to the evidence appraisal of some standard interventions for important somatic disorders (e.g., for hypertension [39] and asthma [40]).

#### Adequacy of blinding: are patients, clinicians, and researchers aware of treatment allocation?

A major potential source of bias and therefore for the quality of RCT evidence relates to the presence and adequacy of blinding—whether patients or assessors of outcomes are aware of who received what intervention. There is potential for unblinding even during well-designed and carefully conducted pharmacological trials, which it has been suggested could undermine confidence in all of the evidence for efficacy (e.g., [36]). While it is possible that the apparent effects of medication could be exaggerated by unblinding due to adverse effects, this has not been demonstrated nor has this view been endorsed by the major evidence-based treatment guidelines, (e.g., [41]). The potential for bias due to lack of blinding is particularly acute for psychosocial interventions where: (i) blinding is very difficult to implement with integrity and (ii) patients/parents are often the informants for the primary outcomes. To address this problem, the European ADHD Guidelines Group (EAGG), a working group of the European Network for Hyperkinetic Disorders (EUNETHYDIS) estimated the impact of blinding on results in their series of meta-analyses of non-pharmacological treatments for ADHD [26, 42–45]. They compared what they termed the “most proximal” outcome (MPROX—i.e., rated by persons closest to treatment delivery and, therefore, the most vulnerable to lack of blinding) with the measure judged by the group consensus to be most blinded (PBLIND, i.e., probably blinded). These comparisons provided a range of estimates of treatment efficacy adjusted for degree of blinding. The EAGG meta-analyses found that: (i) MPROX effects were considerably larger (and more significant) than PBLIND effects; (ii) the scale of this MPROX-PBLIND discrepancy varied by treatment type—largest for parent training (where blinding was most challenging to implement) and smaller (though still substantial) for neurofeedback and cognitive training; (iii) the stronger the study design (e.g., sham/placebo controlled designs), the smaller the discrepancy. Even in apparently well-blinded studies (with a sham control), PBLIND gave smaller effects than MPROX. These findings highlight that optimal assessment of treatment efficacy should combine blinding by design and blinding by reporter. It is, however, important to acknowledge that un-blinded outcome measures may still add value, as they may reflect changes in other aspects of the situation that are clinically relevant but not picked up by the blinded raters who may be evaluating more limited range of behaviors.

#### What are the selection criteria for the clinical sample?

Patient selection entry criteria also represent a potential source of bias and limit generalisability of findings. Most clinicians are aware of the issues relating to non-inclusion in most RCTs of those more complex comorbid cases that are common within their clinical practice. There are, however, several other issues that are not so obvious. For example, many recent medication trials have included enriched samples, for example, excluding participants who have previously not responded to or had adverse effects from one of the study drugs. Although these criteria were implemented for ethical reasons, they are likely to introduce bias, usually in favor of the study drug. Non-pharmacological trials also typically recruit less complex and hence easier to treat patients for reasons of convenience and this can affect trial findings [37]. In non-pharmacological trials, another challenge is that results may be difficult to interpret because of variation in whether participants are receiving ADHD medication, which could reduce the effect of the tested intervention.

#### Have long as well as short-term effects been assessed?

While there is a wealth of data regarding the short-term efficacy of treatments for ADHD, high-quality data on longer term outcomes are still lacking and the long-term effectiveness of ADHD interventions, both pharmacological and non-pharmacological, continues to be debated [28, 46, 47]. The situation has improved somewhat, since the European Medicines Agency (EMA) required companies to include longer term efficacy trials of at least 6 months or with a randomized withdrawal period of at least 6 months as well as long-term safety trials of at least 1 year, as a part of the data presented for marketing authorization (http://bit.ly/1O2XRPp). Several of these randomized discontinuation trials have now been published although few have exceeded the 1 year duration required by the regulatory authorities [48–51].

A number of studies, including the Multimodal Treatment of ADHD (MTA) study [27] and observational cohort studies, provide information about longer term efficacy and adverse effects. While hugely valuable in many respects, the non-random nature of the data limits the conclusions about causal effects. These designs often include a lack of blinding and biases in a number of factors influencing who remains on medication (e.g., [52, 53]).

#### Are adverse effects adequately assessed?

Adequate measurement of short- and long-term adverse effects is often a concern. While adverse events are routinely measured in drug trials which are required to comply with legal and ethical requirements, this has traditionally been through spontaneous self-report and over relatively short time frames. The EMA guidelines on the clinical investigation of medications for ADHD have also insisted that companies conduct open-label safety studies of at least 1 year with prospective follow-up for a longer period of time as a part of the Risk Management Plan (RMP) post-licensing (http://bit.ly/1O2XRPp) and have directed that these studies focus on growth and puberty, cardiovascular safety, as well as psychiatric and neurological adverse effects. However, the lack of a comparison group in these studies (e.g., [54]) makes interpretation of their findings much more complicated. Very few non-pharmacological trials make any attempt to measure possible adverse outcomes. The assumption that such interventions are unlikely to have any negative effects is perhaps naive and until recently rarely tested [55] and the recent work suggests that this may be an under-appreciated phenomenon [56].

#### What can we conclude from observational studies?

Despite their clear limitations [57], observational studies have certain advantages and can helpfully complement RCTs. They importantly often include real-world outcomes, such as criminal convictions [58, 59], violent re-offending [60], depression [61], suicidality [62, 63], substance misuse [64, 65], psychotic disorders and hallucinations in childhood and adolescence [66], childhood injuries [67–69], emergency visits [70–72], and transport accidents [73], motor vehicle crashes [31], and school performance [74, 75]. Further strengths of these studies include their large samples and potential for observation over extended periods. Several studies have adopted innovative designs, such as the use of self-controlled case series, e.g., [71], aimed at decreasing the bias due to the lack of randomization in observational studies, in an attempt to mitigate these limitations. However, it is still important to recognize limitations that cannot be addressed by the self-controlled case-series design, such as the potential for referral bias, lack of control for time-varying confounders [69], and the frequent lack of information on the validity of diagnoses and explanatory variables, when considering the often impressive findings.

#### How do we assess whether one treatment is better than another?

Network meta-analysis (NMA) is a relatively recent development in the field of evidence synthesis that allows comparison among different interventions, to provide a ranking of interventions based on, e.g., efficacy and tolerability. Supported by the WHO, Cochrane, GRADE, and NICE [76], NMAs have several potential benefits [76], including their ability to take into account a much wider range of evidence and make quantitative comparisons between interventions even when they have not been directly compared in head-to-head studies. However, NMA methodology requires a very rigorous and careful approach to ensure accurate and reliable results. A main consideration is the requirement for transitivity (i.e., that effect modifiers do not substantially differ across the included trials). This is currently particularly challenging when trying to combine evidence from pharmacological and non-pharmacological interventions into a single network of a NMA [77].

### A summary of the current evidence

Multiple systematic reviews and meta-analyses have been published assessing the efficacy and/or tolerability of both pharmacological and non-pharmacological treatments for ADHD and these data have been further scrutinized in the development of evidence-based guidelines.

### Medication

#### Efficacy

The most recent and comprehensive appraisal of pharmacological treatments is the network meta-analysis (NMA) conducted by the EAGG, with stringent inclusion/exclusion criteria (such as excluding enrichment designs and RCTs with add-on treatments) as well as including both published and unpublished data. The main findings on efficacy and tolerability are presented in Table 2. These data support general efficacy and tolerability for a broad range of ADHD medications with methylphenidate in children and adolescents, and amphetamines in adults leading the table based on combined efficacy and tolerability profiles. As described above, a significant limitation in our understanding of pharmacological treatments for ADHD relates to the lack of methodologically sound data on longer term effectiveness which therefore remains uncertain. It is, therefore, important that studies with more advanced designs such as long-term randomized discontinuation trials as well as those using within-subject designs through linked registry data and data from electronic medical records are supported and conducted [78].

**Table 2:** Evidence for pharmacological treatments of ADHD

Intervention	Putative mechanism(s)	Evidence for effect on ADHD core symptoms (total) vs placebo^a^	Evidence for effects on other aspects of functioning vs placebo^b^
Dex-Amphetamine (including lisdexamphetamine and mixed amphetamine salts)	Block of the reuptake of norepinephrine, dopamine Release of dopamine from vesicles into synaptic space	*Teacher ratings* – *Clinician ratings* SMD = **1.02 (0.85–1.19)** *Parent ratings* SMD = **1.07 (0.79– 1.36)**	*Clinical global functioning* OR **7.71 (5.52–10.77)**
Atomoxetine	Selective inhibition of presynaptic norepinephrine reuptake (although note that in prefrontal cortex the norepinephrine transported is responsible for dopamine reuptake	*Teacher ratings* SMD = 0.32 (− 0.18 to 0.82) *Clinician ratings* SMD = **0.56 (0.45–0.66)** *Parent ratings* SMD = **0.60 (0.50–0.71)**	*Clinical global functioning* OR **2.28 (1.38–3.76)**
Bupropion	Inhibition of the reuptake of dopamine, serotonin, and norepinephrine	*Teacher ratings* SMD = 0.32 (− 0.43 to 1.07) *Clinician ratings* **SMD = 0.96 (0.22–1.69)** *Parent ratings* SMD = − 0.24 (− 0.92 to 0.44)	*–*
Clonidine	Stimulation of the central alpha-2 adrenergic receptors, increasing noradrenergic stimulation (NE)	*Teacher ratings* – *Clinician ratings* SMD = **0.71 (0.24–1.17)** *Parent ratings* –	*Clinical global functioning* OR 2.78 (0.91–8.53)
Guanfacine	Stimulation of central alpha (2)-adrenergic receptors (more selective than clonidine) increasing noradrenergic stimulation (NE)	*Teacher ratings* SMD = 0.63 (− 0.35 to 1.62) *Clinician ratings* SMD = **0.67 (0.50–0.85)** *Parent ratings* SMD = 0.23 (− 0.45 to 0.90)	*Clinical global functioning* OR **3.63 (2.36; 5.57)**
Methylphenidate	Block of dopamine and norepinephrine reuptake	*Teacher ratings* SMD = **0.82 (0.48– 1.16)** *Clinician ratings* SMD = **0.78 (0.62–0.93)** *Parent ratings* SMD = **0.84 (0.72–0.95)**	*Clinical global functioning* OR **5.57 (3.99–7.79)**
Modafinil	Mechanism unclear but thought to stimulate central histamine, norepinephrine, serotonin, dopamine, and orexin systems	*Teacher ratings* SMD = **0.76 (0.37–1.15)** *Clinician ratings* SMD = **0.62 (0.41–0.84)** *Parent ratings* SMD = **0.46 (0.31–0.61)**	*Clinical global functioning* OR **3.22 (1.91–5.43)**

Data from Cortese et al. Lancet Psychiatry, 2018.

^a^*SMD: standard mean difference, given with 95% confidence intervals*

^*b*^*OR: odds ratio, given with 95% confidence intervals*

Estimates given in bold typeface denote meta-analytic estimates whose 95% confidence intervals are significantly different from a null effect

#### Tolerability and safety

In relation to adverse events and safety, both individual reviews, meta-analysis, and network meta-analysis demonstrate good short-term tolerability of ADHD medications once the events are equally common in treatment and placebo arms are taken into account. Common short-term adverse effects are well described and routine monitoring is part of standard care as discussed in many clinical guidelines [41, 79]. The same challenges described above for identifying longer term effectiveness also apply to longer term adverse events, but are compounded by the potential problems of statistical power when trying to identify rare but important adverse effects such as completed suicide and severe cardiovascular events. The most effective way to provide these data are through observational studies using large registry and research databases that can provide complementary data to that from clinical trials and meta-analyses. It is therefore essential that these studies are supported and conducted.

### Non-pharmacological interventions

Several high-quality systematic reviews and meta-analyses have also investigated the evidence for the efficacy and effectiveness, but not acceptability or adverse events, of non-pharmacological approaches to treating ADHD (Table 3). For behavioral parent training, blinded ratings do not support an effect on core ADHD symptoms but do show a significant increase in positive parenting and reduction in both negative parenting and children’s oppositional behaviors [44]. The evidence around neurofeedback is most hotly contested, with different research groups presenting different arguments and rationales about which studies should be included, which protocols were used to deliver neurofeedback and which outcomes should be considered [80–84]. Positive effects seen with un-blinded outcomes are not sustained when blinded outcomes [43] or sham controls [85, 86] are included in the analyses. While there are studies showing beneficial effects on inattention [85], we recommend that both standard neurofeedback approaches [43] and the more intensive treatments [85] require further evidence and validation before neurofeedback should be considered as standard clinical interventions for ADHD. Cognitive training, defined as “the process of improving cognitive functioning by means of practice and/or intentional instructions”, showed a medium-to-large effect on unblinded outcomes [26, 42]. These effects remained marginally significant for probably blinded outcomes for ADHD symptoms and significant positive effects on laboratory tests of verbal and visual working memory, but, importantly, were not significant for attention and inhibition or academic functions. An overview of systematic reviews of dietary interventions concluded that individual study methods were weak and that different meta-analyses have used very different inclusion and exclusion criteria and that this has resulted in a wide range of estimated effect sizes [45]. There is a small but statistically significant effect on probably blinded ratings for supplementation with free fatty acids, while the evidence to support either restricted elimination diets or elimination of artificial food colors is less certain.

**Table 3:** Evidence for non-pharmacological treatments for ADHD

Intervention	Putative mechanism (s)	Evidence for effect on putative mechanism (s)	Evidence for effect on ADHD core symptoms (total)	Evidence for other aspects
Behavioral parent training				
Behavioral parent training (EAGG) Daley et al. (2014)	Improve parent child interactions Increase positive parenting Reduce negative parenting	PBlind *Positive parenting* SMD = **0.63 (0.47–0.78)** *Negative parenting* SMD = **0.43 (0.24–0.62)**	PBlind SMD = 0.02 (− 0.30 to 0.34)	PBlind *Conduct problems* SMD = **0.31 (0.05–0.57)**
		Unblinded *Positive parenting* SMD = **0.68 (0.27–1.09)** *Negative parenting* SMD = **0.57 (0.37–0.78)**	Unblinded SMD = **0.35 (0.19–0.50)**	Unblinded *Conduct problems* SMD = **0.26 (0.14–0.37)** *Parental self-concept* SMD = **0.37 (0.03–0.70)** *Parental mental health* SMD = **0.09 (− 0.09 to 0.23)** *Social skills* SMD = **0.47 (0.15–0.78)** *Academic achievement* SMD = **0.28 (0.06–0.50)**
Parenting interventions for pre-schoolers Rimestad et al. (2019)	Improve parent child interactions Increase positive parenting Reduce negative parenting	Independent assessor *Negative parenting* SMD = **0.33 (0.13–0.53)** Parent ratings *Negative parenting* SMD = **0.63** **(0.32–0.93)** Follow-up Parent ratings SMD = 0.12 (− 0.01 to 0.24)	Independent assessor *ADHD symptoms* SMD = 0.12 (-0.12 to 0.36) Parent ratings SMD = **0.51 (0.33–0.69)** Follow-up Parent ratings SMD = 0.07 (− 0.01 to 0.15)	Independent assessor *Conduct problems* SMD = 0.31 (− 0.07 to 0.69) Parent ratings SMD = **0.44 (0.17–0.70)** Follow-up SMD = **0.07 (0.01–0.15)**
Cognitive training				
Cognitive Training (EAGG) Cortese et al. (2015)	Improve executive dysfunctions assumed to underpin ADHD behavioral symptoms	*Working memory visual* SMD = **0.47 (0.23–0.70)** *Working memory verbal* SMD = **0.52 (0.24–0.80)** *Inhibition* SMD = 0.07 (-0.15 to 0.28) *Attention* SMD = 0.14 (-0.19 to 0.48)	PBlind SMD = **0.20 (0.01–0.40)**	*Reading* SMD = 0.09 (− 0.09 to 0.27) *Arithmetic* SMD = 0.01 (− 0.13 to 0.11)
			Unblinded SMD = **0.37 (0.09–0.66)**	
Organizational skills interventions Bikic et al. (2017)	Improve organizational skills	Unblinded teacher-reported organizational skills g = **0.54 (0.17–0.91)** Parent-reported organizational skills g = **0.83 (0.32 to 1.34)**	Unblinded teacher-reported inattention symptoms *g* = **0.26 (0.01–0.52)** Parent-reported inattention symptoms *g* = **0.56 (0.38–0.74)**	Unblinded teacher-reported academic performance *g* = **0.33 (0.14–0.51)** Objective assessment GPA ***g* = 0.29 (0.07–0.51)**
Cognitive training Catala-Lopez et al. (2017)	Improve executive dysfunctions supposed to underpin ADHD behavioral symptoms	N/A	Main outcome (treatment response) Clinicians’ ratings OR 0.33 (0.01–5.64) Parents’ ratings: OR 0.21 (0.01–3.33) Teachers’ ratings: N/A	Global functioning OR 0.39 (0.01–5.80)
Neurofeedback				
Neurofeedback EAGG Cortese et al. (2016)	Improve executive dysfunctions supposed to underpin ADHD behavioral symptoms	*Attention* SMD = 0.13 (− 0.09–0.36) *Inhibition* SMD = 0.30 (− 0.10–0.70)	PBlind SMD = 0.15 (− 0.08 to 0.38)	–
			Unblinded SMD = **0.35** **(0.11–0.59)**	
Neurofeedback Catala-Lopez et al. (2017)	Improve brain dysfunctions supposed to underpin ADHD behavioral symptoms	N/A	Main outcome (treatment response) Clinicians’ ratings N/A Parents’ ratings: OR 0.49 (0.04–4.65) Teachers’ ratings: OR 0.68 (0.05–5.37)	Global functioning N/A
Neurofeedback Bussalb et al. (2017)	Improve brain dysfunctions supposed to underpin ADHD behavioral symptoms	N/A	Main outcome (treatment response) Clinicians’ ratings, total: N/A Parents’ ratings: **SE 0.32 (*p* value: 0.013)** Teachers’ ratings, total: SE − 0.11 (*p* value: 0.37)	N/A
Neurofeedback Van Doren et al. (2019)	Improve brain dysfunctions supposed to underpin ADHD behavioral symptoms		Parent rating Inattention (studies with non-active controls) First time point **SMD = 0.38; (95% CI 0.14–0.61)** Follow-up SMD = **0.57 (95% CI 0.34–0.81)** Parent rating Hyperactivity/impulsivity (studies with non-active conditions) First time point **SMD = 0.25; (95% CI 0.05–0.45)** Follow-up SMD = **0.39 (95% CI 0.19–0.59)**	
Dietary interventions				
Restricted elimination diet EAGG Sonuga-Barke et al. (2013)	Reduce the undesirable brain effects of specific diet components	N/A	PBlind SMD = 0.51 (− 0.02 to 1.04)	N/A
			Unblinded SMD: **1.48** **(0.35–2.61)**	
Artificial food color exclusion EAGG Sonuga-Barke et al. (2013)	Reduce the undesirable brain effects of artificial food colors	N/A	PBlind SMD = **0.42 (0.13–0.70)**	N/A
			Unblinded SMD = **0.32** **(0.06–0.58)**	
Supplementation with free fatty acids EAGG Sonuga-Barke et al. (2013)	Produce desirable changes in the brain biochemistry	N/A	PBlind SMD = **0.16 (0.01 to 0.31)**	N/A
			Unblinded SMD = **0.21** **(0.05–0.36)**	
Polyunsaturated fatty acids (or PUFAs) Catala-Lopez et al. (2017)	Changes brain chemistry in regions supposed to be involved in ADHD	N/A	Across raters: OR 2.14 (0.83–5.57)	N/A
Amino acids Catala-Lopez et al. (2017)	Changes brain chemistry in regions supposed to be involved in ADHD	N/A	Across raters: OR 1.19 (0.25–5.71)	N/A
Minerals Catala-Lopez et al. 2017	Changes brain chemistry in regions supposed to be involved in ADHD	N/A	Across raters: OR 2.93 (0.90–10.15)	N/A
Herbal therapy Catala-Lopez et al. (2017)	Changes brain chemistry in regions supposed to be involved in ADHD	N/A	Across raters: OR 0.59 (0.17–1.99)	N/A

Estimates given in bold typeface denote meta-analytic estimates whose 95% confidence intervals are significantly different from a null effect

### International clinical guidelines: key differences

Clinical guidelines are an important mechanism for increasing evidence-based clinical practice, and improving quality of care and resource allocation. Several national guidelines from Europe and North America, compared in Table 4, have published recommendations for the management of ADHD in children and adolescents. These come from distinct cultures with very different healthcare systems, and with varying rates of diagnostic identification, and differing availability of both pharmacological and non-pharmacological interventions. Guidelines also vary in the clinical issues addressed and methods for reviewing evidence. Methodological differences include: the use or not of professional reviewers versus clinical experts; the prioritization of expert opinion versus evidence base; undertaking of an independent primary review of the literature or reliance on existing reviews and whether costs of intervention are considered when making recommendations.

**Table 4:** Current national guidelines for ADHD

	NICE (2018)	German guidelines for ADHD (2018)	Dutch guidelines for ADHD (2019)	Spanish guidelines for ADHD (2017)	CADDRA (2018)	AAP (2019)
Target group	Children, young people and adults	Children, adolescents and adults	Children, adolescents and adults	Children, adolescents and adults	Children, adolescents and adults	Children and adolescents
Who can diagnose and prescribe	Diagnosis by specialist psychiatrist, paediatrician or other appropriately qualified healthcare professional with training and expertise in ADHD diagnosis, working in multi-disciplinary ADHD services. Prescribing can be shared with primary care physician. There should be formal shared care arrangements	Diagnosis by child and adolescent psychiatrist, child psychologist, paediatrician or other appropriately qualified healthcare professional with training and expertise in ADHD diagnosis Prescribing should be by child and adolescent psychiatrist or paediatrician	Diagnosis by a trained and qualified healthcare professional; this is in general a psychiatrist or psychologist, or paediatrician, and in some instances a general practitioner, all with appropriate training in diagnosing ADHD and related conditions Prescribing can in principle be done by every physician but in practice should be done only by physicians with appropriate training and clinical experience, thus psychiatrists or paediatricians; general practitioners are not expected to initiate prescriptions but can continue prescriptions initiated by experts, and if needed, seek advice from experts. The same applies to nurse practitioners who can continue prescriptions initiated by experts	Professional with specific training to assess comorbidities and Differential diagnosis. Paediatrician, Psychologist, Child & Adolescent Psychiatrist (CAP) (or General Psychiatrist for adults) and Paediatric Neurologist can diagnose Prescribing limited to family doctor, primary care physician, paediatrician or psychiatrist	Fully licensed and adequately trained clinicians	These guidelines are addressed to paediatricians or other primary care clinicians (PCCs) and state that PCCs may benefit from additional consultative support and guidance from child and adolescent psychiatrists, clinical child psychologists, developmental/behavioral paediatricians, neurodevelopmental disability physicians, child neurologists, or child- or school-based evaluation teams These guidelines provide recommendations on the prescription for PCCs
Domains and interventions included in guidelines	Assessment^1^ & Diagnosis/Recognition Management Information & support Psychological interventions Medication and monitoring Combining psychological interventions and medication Interventions in educational settings^1^ Dietary interventions^2^ Transition^1^ Organisation of care^1^	Assessment & Diagnosis Treatment planning Psychosocial interventions (parent, patient, school-based) Medication Neurofeedback Dietary interventions Inpatient treatment, rehabilitation & youth welfare services Transition Self-help	Screening/prevention Assessment/diagnosis/monitoring Treatment Participation/re-integration Patient perspective Organisation of care	Focus on Interventions: Psychosocial interventions Pharmacological interventions Organizational and life skills support and training	Assessment & Diagnosis Psychosocial interventions Medication Neurofeedback Dietary interventions	Assessment & Diagnosis Psychosocial interventions Medication and monitoring Interventions in educational settings Organisation of care Implementation
Independent systematic review of evidence (yes/no)	Yes	Yes	Yes (based on 2018 NICE REF search but reorganized/re-done and updated with individual studies when evidence was lacking)	Yes (Also considered other Guidelines: NICE REF, American Academy of Paediatrics REF)	Partly for treatment section (=based on published evidence & expert consensus when lack of evidence)	Yes (but the search, which was conducted by the US Agency for Healthcare Research and Quality (AHRQ) was limited to research published before 2016)
How evidence evaluated	Systematic review/meta-analysis, Grade	Systematic review/AWMF/Grade	Systematic review/meta-analysis, Grade	Evidence level and degree of recommendation SIGN REF Other guidelines’ recommendations scored using AGREE (Appraisal of Guidelines Research and Evaluation)	In part: systematic review	Systematic review with evidence graded using AAP policies
Recommendations based on ADHD severity (Y/N)	No	Yes	Y	Y	No	No
Recommendations vary according to age (Y/N) and age groups	Yes	Yes	Yes	Yes	Yes	Yes
First line treatment recommendation (med or non-med)	Children < 5 years: ADHD-focused group parent training. Medication treatment not recommended School-aged children: 1st ADHD-specific information & support. 2nd If persistent & significant impairment in at least one domain of life: offer medication If comorbid oppositional defiant disorder or conduct disorder: add in a parent training programme For adolescents: 1st Medication 2nd If symptoms still impairing in at least one domain of life after medication treatment: offer cognitive behavioral therapy	Medication for moderate to severe ADHD (age 6-18 years); in severe cases for children aged 4-6 after psychological treatment Psychological treatment for children younger than 6 years old and for children and adolescents aged 6 to 18 years with mild to moderate ADHD	Children <6 years: 1st Parent/teacher training; medication only considered in case of non-response [NR] to parent/teacher training 6-12 years: -Those with behavioral problems + mild–moderate ADHD 1st parent–teacher training + Severe ADHD 1st shared decision-making medication or parent/teacher training; In case of NR the other option -Those without behavioral problems + mild ADHD: 1st parent–teacher training + Moderate–severe ADHD: Shared decision-making medication or parent/teacher training; In case of NR other treatment option 13–18 years: -Without/with behavioral problems + ADHD mild: 1st CBT with adolescent and parent/teacher involvement + ADHD moderate–severe: shared decision-making CBT with adolescent and parent/teacher involvement or medication; in case of NR other treatment option + ODD/CD diagnosis also follow guidelines ODD/CD	Children <5 years: Medication not recommended School-aged children and adolescents: 1st Psychological of pedagogical treatment/academic support 2nd Medication only recommended if 1st does not work, or in severe cases. Adults: 1st pharmacological treatment in moderate to severe cases. Optional to use psychosocial OR medication in mild cases	Psychosocial interventions for pre-schoolers	For pre-school children (4-–6 years): Parent and/or teacher-administered behavior therapy as the first line of treatment For children 6–11 years of age: FDA-approved medications for ADHD and/or evidence-based parent- and/or teacher-administered behavior therapy as treatment for ADHD (preferably both) For adolescents 12–18 years of age: FDA-approved medications for ADHD with the assent. Evidence-based training interventions and/or behavioral interventions as treatment of ADHD, if available
Universal recommendation for Psychological intervention (Y/N) and qualifications – for what, how delivered, e.g., group/individual, timing, etc	Yes School-aged children and adolescents: ADHD-specific information & support with review prior to considering medication	Yes Psychoeducation; Group based or individual, parent and/or teacher and/or patient based psychological treatment	Yes Psychoeducation & advice to parents/teachers Preference parent leading for individual or group-based intervention Supervision, training and evidence-based program recommended	Yes Organizational skills training, Psychoeducation, School/Health Care System/Family coordination, Social skills, Executive function training, Cognitive–Behavioral Therapy	Delivery format not specified	Yes Psychoeducation to child and family. Delivery format not specified. Educational interventions and individualized instructional supports are considered a necessary part of the treatment plan
Sequencing of medication	Yes School-aged children and adolescents: 1st methylphenidate, 2nd lisdexamfetamine, (consider dexamphetamine if lisdexamfetamine not well tolerated), 3rd atomoxetine or guanfacine	Yes 1st stimulants 2nd atomoxetine or guanfacine ADHD + Anxiety: stimulants or atomoxetine ADHD + substance misuse: long-acting stimulants or atomoxetine or guanfacine ADHD + Tics: stimulants or atomoxetine or guanfacine	Yes 1st methylphenidate 2nd dexamphetamine 3rd atomoxetine or guanfacine	No Medications recommended (no order specified): methylphenidate, lisdexamfetamine, guanfacine and atomoxetine	Yes 1st long-acting stimulants 2nd Atomoxetine, Guanfacine XR and short/intermediate acting psychostimulants 3rd bupropion, clonidine, imipramine and modafinil	Yes (implicitly) “the evidence is particularly strong for stimulant medications and sufficient but less strong for atomoxetine, extended-release guanfacine, and extended-release clonidine (in that order)”
Health economics included	Yes	No	Yes	No	No	No
Patient/caregiver opinion included in Guideline	Yes	Yes	Yes	Yes	No	Yes

Balance between evidence- and Opinion-led:

1= exclusively or almost exclusively evidence-based

2= mainly evidence-based but expert opinion where absence of evidence

3= mainly expert opinion

^1^2008 NICE Guideline only REF

^2^2016 NICE Guideline Addendum REF

The recommendations of the cited guidelines (apart from the Canadian CADDRA guidelines which did not include a systematic review of the evidence) were developed by combining evidence summaries (based on systematic reviews, meta-analyses, and assessment of quality of evidence, with the most recent guidelines using the GRADE system for evaluating quality of evidence [35]), with expert opinion.

Despite being largely reliant on the same evidence base, differences in recommendations are especially likely when high-level evidence (i.e., meta-analyses randomized-controlled trials; RCTs) is lacking. In such circumstance, different groups have taken different approaches—the UK NICE [87] and the German Guidelines [88] use expert opinion, the Dutch Guidelines [89] take individual studies into account, and the Spanish Guidelines [90] consider the recommendations of other guidelines including NICE and the American Academy of Pediatrics [91], evaluated using the Appraisal of Guidelines for Research and Evaluation (AGREE) methodology [92]. Only NICE and the Dutch Guidelines included health economic evidence when considering their recommendations.

### Similarities across guidelines

In summary, in relation to assessment and diagnosis

- All guidance specifies who can diagnose and prescribe; European (UK, German, Dutch and Spanish) guidance makes specific reference to specialist ADHD training among paediatricians and psychiatrists, while North American guidance includes primary care physicians. For prescribing, UK NICE guidance also allows shared care plans between specialists and primary care physicians.
- All guidelines agree that the clinical interview and direct observations, including assessments of impairment, physical and psychiatric comorbidity, and family history, provide the basis to establish a diagnosis.
- Rating scales are recommended as auxiliary diagnostic tools and as systematic outcome measures in treatment monitoring, rather than as the gold standard for the diagnosis.
- Neuropsychological assessment is not considered to be essential to establish the diagnosis, even though it can provide useful information to better tailor the management.
- In relation to treatment:
- All guidelines take account of developmental differences, but some specifically stratify treatment recommendations according to age, with three groups identified:
  o *Young children*: under 5 years (NICE) or under 6 years (German and Spanish Guidelines); *school-aged children*: 5/6-18 years; and *adults*: >18 years. The Dutch guidelines further differentiated between children 6 and 12 and adolescents 12 and 18 years.
- The RCT evidence for efficacy and safety of medication in young children is considered too limited to recommend medication for *routine* treatment in this age group, but medication is an option that could be considered in selected cases.
- When pharmacological treatment is indicated, most guidelines suggest that stimulants are the preferred first-line medication. The Spanish Guidelines only indicate that any of the approved medications can be used without offering any recommended order of priority.
- All agree on the need for psychosocial interventions, but differ in their timing in the management of ADHD. In contrast to the other guidelines, the NICE 2018 Guideline no longer recommends parent training as first-line treatment in school-aged children because of the lower effect sizes and poorer quality of evidence compared to medication, but ADHD-focused parent training remains the first-line intervention for pre-school children.

Guidelines differ in whether they consider ADHD severity as a factor when making recommendations. NICE (2018) currently do not, while the German and Dutch Guidelines, adopting the DSM-5 definitions of severity, do. Both the Dutch and NICE guidelines also take co-occurring ODD/CD into account. As the main aim of the severity specifier is to avoid overuse of medication in less severe cases, these different approaches may reflect variation in administrative diagnostic rates, which, for example, are higher in Germany (3.2–6.1%) [93–95] than in the UK (0.5–1.5%) [96]. In the Spanish Guidelines, where children are severely affected, clinicians may prescribe outside the formulary (i.e., use medication in children <6 years old).

Apart from the consensus-based CADDRA guidelines [97], these guidelines include a few specific recommendations on personalized approaches to ADHD interventions, including co-occurring conditions, and where these occur, they rely largely on expert opinion, rather than trial evidence.

With respect to pre-medication investigations (Table 5), all guidelines take an individualized approach, based on personal and family history of possible cardiac disease together with essential physical assessments including height, weight, blood pressure, and pulse. None of the guidelines require routine cardiac investigations, such as an electrocardiogram. For all guidelines, ongoing monitoring includes height, weight, pulse, and blood pressures, as well as a systematic review of benefits and possible adverse effects. Guidance varies in the extent it specifies the enquiry around class-specific possible adverse effects.

**Table 5:** Guideline recommendations on investigations prior to initiating medication and ongoing monitoring during treatment

	NICE (2018)	German guidelines for ADHD (2018)	Dutch guidelines for ADHD (2019)	Spanish guidelines for ADHD (2017)	CADDRA (2018)	AAP (2019)
Pre-medication checks	Medical history relevant to possible drug contraindications Height, weight, pulse, blood pressure Cardiology referral or ECG only under specified circumstances (history or clinical signs of cardiac disease; family history of cardiac disorder)	Medical history relevant to possible drug contraindications Height, weight, pulse, blood pressure Cardiology referral or ECG only under specified circumstances (history or clinical signs of cardiac disease; family history of cardiac disorder)	Medical history relevant to possible drug contraindications Height, weight, pulse, blood pressure Cardiology referral and/or ECG only under specified circumstances (history or clinical signs of cardiac disease; family history of cardiac disorder)	Height, weight blood pressure and pulse Medical evaluation to rule out medical causes of inattention, hyperactivity and impulsivity	Weight, height, blood pressure, heart rate, history of light headedness, shortness of breath, or other possible cardiac symptoms, and any family history of suspected cardiac sudden death	Child or adolescent’s history of specific cardiac symptoms in addition to the family history of sudden death, cardiovascular symptoms, Wolff–Parkinson–White syndrome, hypertrophic cardiomyopathy, and long QT syndrome
Ongoing monitoring for all medication classes	Systematic review of systems Assess symptom change Height, weight, pulse and blood pressure	Systematic review of systems Assess symptom change Height and weight, pulse and blood pressure	Systematic review of systems Assess symptom change Height and weight pulse and blood pressure	Weight, height, Blood pressure and pulse monitoring is recommended to assess possible cardiovascular & growth effects of medication	Height and weight in children New mood, anxiety, substance use disorder, psychotic or manic symptoms Suicidal behavior or ideation Aggressive behavior (new or worsening) Sleep, appetite Irritability/mood swings	Height, cardiovascular parameters (mentioned, not specific recommendation)
Class-specific ongoing monitoring						
Stimulants	Tics Potential for stimulant diversion	Tics Potential for stimulant diversion	Tics Potential for stimulant diversion	Not mentioned	BP, HR (may increase) Priapism Growth retardation Peripheral vasculopathy including Raynaud’s Phenomenon	Appetite loss, abdominal pain, headaches, and sleep disturbance. Decreasing growth velocity, hallucinations and other psychotic symptoms
Atomoxetine	Sexual dysfunction	Sexual dysfunction		Not mentioned	Priapism and urinary retention Signs/symptoms of liver injury Growth retardation Peripheral vasculopathy including Raynaud’s Phenomenon	Initial somnolence and gastrointestinal tract symptoms, particularly if the dosage is increased too rapidly, and decreased appetite. Less commonly, an increase in suicidal thoughts, Extremely rarely, hepatitis
Guanfacine		Sedation, somnolence, fatigue	Sedation, somnolence, fatigue	Not mentioned	Somnolence and sedation BP, risk of hypotension Bradycardia, syncope Elevated BP and HR upon abrupt discontinuation QTc interval (to be monitored if underlying conditions or other medication increase the risk of prolonged QTc interval)	Somnolence, dry mouth, dizziness, irritability, headache, bradycardia, hypotension, and abdominal pain

### Translating the evidence and guidance into day-to-day management of ADHD

Careful and close application of evidence-based protocols, such as those employed in the MTA trial, often lead to better outcomes for patients in terms of ADHD symptoms [98] and there is emerging evidence that these improvements can be translated into routine practice [99]. While the use of evidence-based guidelines to inform the delivery of treatment is strongly recommended, guidelines usually focus on applying evidence from RCTs at the group level in a probabilistic way to inform decision-making for individual cases. While this is a core principle of evidence-based practice, there are clearly other factors that need to be considered alongside the evidence and guideline recommendation. These more general ‘management’ issues are often less well researched and not always as well covered by published guidelines. While it is tempting to rely solely on effect sizes when comparing the possible value of different treatment approaches for a particular patient, it is also important to take into account the methodological limitations described above when considering comparisons between different treatments. It is also important to consider that: (1) different treatment approaches have different mechanisms of action and that these can impact on factors such as duration of action or acceptability and (2) different treatments also target different areas of impairment. The contrasts between different approaches are important to consider both when deciding between pharmacological and non-pharmacological approaches and also *within* each approach, selecting the interventions most suitable for individual patients. While all of the licensed ADHD drugs primarily target a reduction in ADHD symptoms, impact on other symptoms such as emotional dysregulation and mind wandering as well as indirect effects on associated mood [100], conduct problems [101], and other functional domains [102] have been demonstrated.

In terms of duration of action, while the non-stimulants atomoxetine and guanfacine have lower effect sizes on ADHD core symptom reduction [103], they have a longer duration of action than stimulants, which may be particularly relevant for some patients (such as those with long-day schedules). Parent training, on the other hand, is useful in modifying longer term parent–child interactions, such that there is not only a long-term change in parenting style but also in a child’s behavior; because these are common problems in ADHD, guidelines have retained parent training as a recommended treatment. While still controversial interventions, both neurofeedback and cognitive training also seek to make long-term changes to brain functioning that go beyond symptom control [104]. The issue for both is whether the observed changes in laboratory measures of brain activity and cognition translate to improved symptoms and/or functioning in everyday life. The ‘or’ is important in view of several studies that have indicated a dissociation between improvement in cognition or brain function and symptoms [105].

*Patient and parent priorities;* It is important to ensure that patient and parental priorities, preferences and choices are taken into account through the provision of information, followed by a process of shared decision-making [106]. Families will have their own concerns and preferences [107], which may be influenced by personal experience, other family members, or information from the wider public or accessed through the media, which can often be inaccurate and contradictory [108, 109]. It is particularly important to seek ways to include children and young people in decisions about their treatment, and focus on enabling them to take increasing levels of responsibility for selecting, adhering to, and monitoring their care as they develop [106]. Young people must be involved in discussions around transition to adult services [110, 111].

Much of the broader management aims for ADHD can be achieved through high-quality psychoeducation offered at the beginning of the treatment process and topped up at intervals thereafter [112]. Evidence-based psychoeducation should help families and young people become more knowledgeable about ADHD and co-occurring conditions and their management, considering of their individual, familial, and cultural health beliefs and constructs, and their concerns, attitudes, and beliefs about ADHD. It should empower them as consumers to take active informed responsibility for their treatment plan and enhance therapeutic adherence [113].

After psychoeducation, a shared management plan should be created, prioritizing problems and translating them into aims and action plans. Parents and children may have different priorities for treatment targets and these differences should be identified and discussed to build consensus and focus [106].

#### Monitoring to optimize outcomes

While we strongly support treatment optimization and the use of measurement-based care [114], there are few data on ADHD to guide best practice in this respect. Measurement-based care, aimed at reducing symptoms and identifying adverse events, can improve both short- and long-term clinical outcomes and enhance treatment adherence [99]. It is, however, essential that clinicians also focus on broader outcomes when monitoring care, including quality of life [115] as well as patient/family priorities. For those not achieving optimal outcomes from the first treatment/s, alternative approaches should be considered [116]. While the sequencing of treatment options [25, 117, 118] has received some attention, a few studies have looked at more general management considerations. Stepped care approaches which present different treatment approaches of increasing intensity dependent on response have been discussed for many years but have not yet been well studied. Several well-designed studies examining various stepped care approaches for ADHD are underway, which, when completed, should give new insights into this approach. [119–122].

#### Treating ADHD in the presence of co-occurring disorders

Co-occurring disorders commonly present a complicating factor when managing ADHD [123]. In prioritizing and sequencing treatment decisions, consideration should be given to which condition is causing the most (acute) distress, which has the most effective interventions, and whether treatment of one is needed before intervention for another is likely to be effective [124–126]. There is also evidence that for some co-occurring disorders, integrated treatment for both at the same time is the most effective approach (e.g., ADHD and anxiety [127], ADHD, and substance use disorder [128]). There can be important interplay between treatments. These are not limited to drug interactions, which always need to be considered, but might also include interactions between pharmacological and psychological treatments such as reduced medication dose following behavioral treatments [117, 129–131].

### Implications for service organization

#### Multi-disciplinary collaboration

When developing clinical services for children and adolescents with ADHD, a multi-disciplinary team is preferable to facilitate comprehensive evaluations including comorbidities, and to provide a wide range of therapeutic options [124, 132]. Patients with ADHD often present with multiple impairments and benefit not only from the interventions provided by psychiatrists and psychologists that target core symptoms, but also from the skills of speech and language therapists, nurses, occupational therapists, psychotherapists, and family therapists, who focus on associated conditions, on treatment adherence and the challenges of ADHD for the family. Clinicians should also promote interventions and environmental modifications that increase responsibility and social participation more widely. This includes supporting schools to utilize effective management strategies [133–135] and also promoting ways that the community can be more broadly engaged and supported to help those with ADHD achieve better integration and involvement [136]. Community-based staff are well placed to support lines of communication with educational and social care agencies.

#### Strengthening care pathways

Clinicians and commissioners of services need to consider and seek to reduce many barriers that limit access to ADHD care [137, 138]. These include: limited provision of services; inadequate recognition of ADHD as a potential cause of impairment and distress; complex referral pathways and mechanisms for financial reimbursement. A chronic shortage of appropriately trained health professionals within the workforce also reduces access to the full range of appropriate evidence-based interventions [139, 140].

#### Service innovation

The dominant model of care is provision in a clinical setting, one-on-one, with highly trained professionals. However, this model can be expensive and limit access [136]. Non-clinical services and combined mental/physical health services may be helpful in the management of young people with ADHD, the delivery of interventions, and reaching those families that have difficulty accessing regular clinical services [136, 141–143]. Other non-clinical community settings including charities and support groups as well as online resources and (partly) digital interventions can increase capacity and lower costs without sacrificing effectiveness [136, 142]. These approaches may be particularly appropriate for interventions such as parent training, CBT, psychoeducation, and coaching [143]. The COVID-19 pandemic has accelerated the implementation of online, remote assessments and particularly interventions for ADHD. While necessary during recent periods of social restriction, these have potential longer term utility to increase access for those living at a distance or otherwise finding it difficult to attend in person. It will be important to ensure that other social groups are not excluded by these innovations. School-based interventions are associated with moderate-to-large functional improvements in meta-analyses [144], although not in all studies [145].

## Conclusions

ADHD is the most common neurodevelopmental disorder in children and adolescents. Although ADHD presents with varying levels of severity, a substantial proportion of affected individuals experience persistent symptoms and substantial impairment from their core ADHD and/or co-occurring symptoms. Therefore, the accurate identification of ADHD and implementation of effective interventions is essential. There is a substantial evidence base, at least in the short term, for a range of pharmacological and, for non-core symptoms, some non-pharmacological treatments, with considerable consensus across different national guidelines emanating from societies that are diverse in terms of their rates of acceptance and identification and their organization of healthcare. However, as in many areas of healthcare, there are significant methodological problems with much of the research underpinning these guidelines and the clinicians should be aware of these and how they may limit interpretation of the evidence. Despite these methodological concerns, clinicians should be confident that ADHD medications are effective, well-tolerated, and safe. Parent training provides an important complement, but largely in relation to reducing additional behavioral problems and improving parent–child relationships. High-quality evidence for neuromodulation—including neurofeedback and cognitive training—is more limited, and as a consequence, these should not currently be recommended as the first-line interventions for core symptoms.

Management of ADHD extends beyond the implementation of guidelines and should be seen as a partnership with patients and their parents, underpinned by psychoeducation and a shared, agreed-upon management plan that considers individual treatment priorities and preferences. While standard care is delivered in clinical settings through one-to-one patient/clinician relationships, schools and third sector settings have much to contribute to a comprehensive approach. Future research should evaluate the utility of digital methods for monitoring outcomes and delivering psychological interventions [146], but it will also be important to understand what accommodations are most effective at helping patients and their families’ access and engage with care.

In summary, ADHD is an important disorder to treat and manage over time, in relation to core symptoms, co-occurring problems, and improvement of real-world outcomes. Clinicians should be robust in expecting substantial improvement with evidence-based treatment but should undertake this in partnership and with a focus on outcomes that are meaningful for patients.

## Data Availability

This guidance represents a synthesis of previously collected data. There are no previously unpublished data included.
